# Supplementary material for: Serological fingerprints link antiviral activity of therapeutic antibodies to affinity and concentration
Source: Sci Rep. 2022 Nov 17;12:19791. doi: 10.1038/s41598-022-22214-z (PMC9672333; doi:10.1038/s41598-022-22214-z)
Supplement: Supplementary file 1 — Supplementary Information. [file 41598_2022_22214_MOESM1_ESM.pdf]

Supplementary Information

Serological fingerprints link antiviral activity of therapeutic antibodies to affinity and concentration

Sebastian Fiedler[1], Sean R. A. Devenish[1], Alexey S. Morgunov[1,2], Alison Ilsley[1], Francesco Ricci[1], Marc Emmenegger[3], Vasilis Kosmoliaptsis[4,5,6], Elitza S. Theel[7], John R. Mills[8,9], Anton M. Sholukh[10], Adriano Aguzzi[3], Akiko Iwasaki[11,12,13,14], Andrew K. Lynn[1], Tuomas P. J. Knowles[1,2,15*]

[1]Fluidic Analytics, Unit A, The Paddocks Business Centre, Cherry Hinton Road, Cambridge CB1 8DH, United Kingdom

[2]Centre for Misfolding Diseases, Yusuf Hamied Department of Chemistry, University of Cambridge, Lensfield Road, Cambridge CB2 1EW, United Kingdom

[3]Institute of Neuropathology, University of Zurich, 8091 Zurich, Switzerland

[4]Department of Surgery, University of Cambridge, Addenbrookes Hospital, Cambridge, CB2 0QQ, United Kingdom

[5]NIHR Blood and Transplant Research Unit in Organ Donation and Transplantation, University of Cambridge, Hills Road, Cambridge CB2 0QQ, United Kingdom

[6]NIHR Cambridge Biomedical Research Centre, Hills Road, Cambridge CB2 0QQ, United Kingdom

[7]Division of Clinical Microbiology, Department of Laboratory Medicine and Pathology, Mayo Clinic, Rochester, Minnesota, USA

[8]Department of Laboratory Medicine and Pathology, Mayo Clinic, Rochester, Minnesota, USA

[9]Center for MS and Autoimmune Neurology, Mayo Clinic, Rochester, Minnesota, USA

[10]Vaccine and Infectious Disease Division, Fred Hutchinson Cancer Research Center, Seattle, Washington, USA

[11]Department of Immunobiology, Yale School of Medicine, New Haven, CT 06519, USA

[12]Department of Epidemiology of Microbial Diseases, Yale School of Public Health, New Haven, CT 06510, USA

[13]Department of Molecular, Cellular and Developmental Biology, Yale University, New Haven, CT 06511, USA

[14]Howard Hughes Medical Institute, Chevy Chase, MD 20815, USA

[15]Cavendish Laboratory, Department of Physics, University of Cambridge, JJ Thomson Ave, Cambridge CB3 0HE, United Kingdom

*to whom correspondence should be addressed: tpjk2@cam.ac.uk

Table S1. Mutations within the spike protein shared among SARS-CoV-2 variants[44].

		SARS-CoV-2 variant										
Spike protein		Alpha	Beta	Gamma	Delta	Kappa	Omicron (BA.1)	Omicron (BA.2)	Eta	Iota	Lambda	Mu
Domain	Region	Shared mutations										
S1	NTD	L18F	L18F									
					T19R			T19I				
				P26S				P26-				
							A67V		A67V			
		H69-					H69-		H69-			
		V70-					V70-		V70-			
							T95I			T95I		T95I
							G142-	G142-				
							V143-	V143-				
		Y144-					Y144-	Y144-	Y144-			Y144S
							Y145D	Y145D				Y145N
										D253G	D253N	
	RBD						G339D	G339D				
							S371L	S371F				
							S373P	S373P				
							S375F	S375F				
			K417N	K417T			K417N	K417N				
							N440K	N440K				
					L452R	L452R					L452Q	
							S477N	S477N				
					T478K		T478K	T478K				
			E484K	E484K		E484Q	E484A	E484A	E484K	E484K		E484K
							Q493R	Q493R				
							Q498R	Q498R				
		N501Y	N501Y	N501Y			N501Y	N501Y				N501Y
							Y505H	Y505H				
	SD2	D614G	D614G	D614G	D614G	D614G	D614G	D614G	D614G	D614G	D614G	D614G
				H655Y			H655Y	H655Y				
							N679K	N679K				
		P681H			P681R	P681R	P681H	P681H				P681H
S2			A701V							A701V		
							N764K	N764K				
							D796Y	D796Y				
	HR1				D950N							D950N
							Q954H	Q954H				
							N969K	N969K				

Table S2. Mutations within the spike protein unique among SARS-CoV-2 variants[44].

		SARS-CoV-2 variant										
		**Alpha**	**Beta**	**Gamma**	**Delta**	**Kappa**	**Omicron (BA.1)**	**Omicron (BA.2)**	**Eta**	**Lota**	**Lambda**	**Mu**
		**Unique mutations**										
		A570D	D80A	T20N	E156-	E154K	N211-	L24-	Q52R	L5F	G75V	R346K
		T716I	D215G	D138Y	F157-	Q1071H	L212I	P25-	Q677H		T76I	
		S982A	L241-	R190S	R158G		G446S	A27S	F888L		R246-	
		D1118H	L242-	T1027I			G496S	V213G			S247-	
			A243-	V1176F			T547K	T376A			Y248-	
							N856K	D405N			L249-	
							L981F	R408S			T250-	
											P251-	
											G252-	
											F490S	
											T859N	

Table S3. Summary of unpublished microfluidic antibody affinity profiling data from SARS-CoV-2 convalescent samples shown in Figure 3. Results from samples collected by Blutspendedienst (BDS) Kanton Zürich and University Hospital Zurich (CH) have been published previously in *Life Sci. Alliance* 2021, 5 (2), e202101270.

**Sample source: Working reagent for anti-SARS-CoV-2 immunoglobulin, NIBSC code 21/234. National Institute for Biological Standards and Control (UK)**							
	**K_D (nM)**				**[antibody] (nM)**		
**antigen**	**best fit**	**lower 95% CI**	**upper 95% CI**		**best fit**	**upper 95% CI**	**lower 95% CI**
SARS-CoV-2 wt spike RBD	12.3	9.3	16.4		110	130	93.9
SARS-CoV-2 delta spike RBD	17.5	13	24.3		119	138	98.1
SARS-CoV-2 omicron spike RBD	30.1	23	39.9		59.3	71.7	46.4
**Sample source: Fred Hutchinson Cancer Research Center, Seattle WA, USA**							
	**K_D (nM)**				**[antibody] (nM)**		
**antigen**	**best fit**	**lower 95% CI**	**upper 95% CI**		**best fit**	**lower 95% CI**	**upper 95% CI**
SARS-CoV-2 wt spike RBD	11.2	3.2	26.8		246	76.7	452
SARS-CoV-2 wt spike RBD	9.3	4.1	18.9		70.5	40.5	110
SARS-CoV-2 wt spike RBD	6.9	3.6	12.2		676.8	597	813
SARS-CoV-2 wt spike RBD	9.4	5.8	13.2		240	196	281
SARS-CoV-2 wt spike RBD	17.4	12.4	23.1		495	414	583
SARS-CoV-2 wt spike RBD	8.6	3.2	25.9		424	230	777
SARS-CoV-2 wt spike RBD	40.4	23.5	64.9		239	161	324
SARS-CoV-2 wt spike RBD	15.1	0.29	37.0		104	3.1	202
SARS-CoV-2 wt spike RBD	4.5	0.01	11.2		190	67.7	349
SARS-CoV-2 wt spike RBD	10.9	3.9	27.4		170	77.3	275
SARS-CoV-2 wt spike RBD	9.3	0.06	28.5		71.7	22.0	127
SARS-CoV-2 wt spike RBD	5.8	1.9	11.0		140	99.6	192
SARS-CoV-2 wt spike RBD	25.4	11.9	60.9		249	127	400
SARS-CoV-2 wt spike RBD	9.7	0.01	1108		9.7	0.09	1416
SARS-CoV-2 wt spike RBD	9.4	3.4	16.1		1778	1176	2270
SARS-CoV-2 wt spike RBD	30.7	11.8	62.5		275	146	788
SARS-CoV-2 wt spike RBD	6.8	1.4	19.6		26.7	11.3	46
SARS-CoV-2 wt spike RBD	48.3	1.0	278		326	4.5	804
SARS-CoV-2 wt spike RBD	9.2	0.01	294		373	0.0	3441
SARS-CoV-2 wt spike RBD	6.5	2.8	11.7		135	108	178
SARS-CoV-2 wt spike RBD	51.8	20.0	124		340	103	1014
SARS-CoV-2 wt spike RBD	16.3	10.5	24.1		297	224	383
SARS-CoV-2 wt spike RBD	7.4	0.02	14.3		96.0	32.0	151
SARS-CoV-2 wt spike RBD	29.2	17.2	47.7		430	241	583
SARS-CoV-2 wt spike RBD	18.5	8.1	37.7		92.0	39.0	153
**Sample source: Mayo Clinic, Rochester, MN, USA**							

	K_D (nM)				[antibody] (nM)		
**antigen**	**best fit**	**lower 95% CI**	**upper 95% CI**		**best fit**	**lower 95% CI**	**upper 95% CI**
SARS-CoV-2 wt spike RBD	0.55	0.011	1.5		2.3	1.2	5.6
SARS-CoV-2 wt spike RBD	0.02	0.010	1.98		22.3	14.6	66.5
SARS-CoV-2 wt spike RBD	1.0	0.013	2.8		19.4	13.1	26.2
SARS-CoV-2 wt spike RBD	0.02	0.010	0.67		3.3	2.0	10.4
